# CD117^+^ cells in the circulation are predictive of advanced prostate cancer

**DOI:** 10.18632/oncotarget.2796

**Published:** 2014-12-17

**Authors:** Bethany A. Kerr, Ranko Miocinovic, Armine K. Smith, Xiaoxia Z. West, Katherine E. Watts, Amanda W. Alzayed, Joseph C. Klink, Maria C. Mir, Tiffany Sturey, Donna E. Hansel, Warren D. Heston, Andrew J. Stephenson, Eric A. Klein, Tatiana V. Byzova

**Affiliations:** ^1^ Department of Molecular Cardiology, Joseph J. Jacobs Center for Thrombosis and Vascular Biology, Lerner Research Institute, Cleveland Clinic, Cleveland, OH 44195, USA; ^2^ Glickman Urological and Kidney Institute, Lerner Research Institute, Cleveland Clinic, Cleveland, OH 44195, USA; ^3^ Department of Anatomic Pathology, Lerner Research Institute, Cleveland Clinic, Cleveland, OH 44195, USA; ^4^ Taussig Cancer Center, Lerner Research Institute, Cleveland Clinic, Cleveland, OH 44195, USA; ^5^ Department of Cancer Biology, Lerner Research Institute, Cleveland Clinic, Cleveland, OH 44195, USA

**Keywords:** CD117/c-kit, CD133, prostate cancer, tumor progression

## Abstract

Circulating tumor cells (CTCs) are associated with cancer progression, aggressiveness and metastasis. However, the frequency and predictive value of CTCs in patients remains unknown. If circulating cells are involved in tumor aggressiveness and metastasis, then cell levels should decline upon tumor removal in localized cancer patients, but remain high in metastatic patients. Accordingly, proposed biomarkers CD117/c-kit, CD133, CXCR4/CD184, and CD34-positive cell percentages in the blood of patients undergoing radical prostatectomy for localized cancer were assessed by flow cytometry prior to intervention and 1–3 months postoperatively. Only circulating CD117^+^ cell percentages decreased after radical prostatectomy, increased with cancer progression and correlated with high PSA values. Notably, postoperative CD117^+^ levels did not decrease in patients experiencing biochemical recurrence. In a xenograft model, CD117-enriched tumors were more vascularized and aggressive. Thus, CD117 expression on CTCs promotes tumor progression and could be a biomarker for prostate cancer diagnosis, prognosis, and/or response to therapy.

## INTRODUCTION

Proposed markers distinguishing prostate cancer from benign tissues include: CD117, CD133, CXCR4, and CD34. [1–3] Specifically, CD133/prominin-1 and CD117/c-kit were documented in multiple solid neoplasms, [2–6] where immunohistological staining of these markers correlated with aggressive tumors and increased resistance to chemotherapy and radiotherapy. [5] Prostate cancers also express CXCR4/CD184, which stimulates metastasis towards the bone microenvironment. [1, 7] CD34^+^ cells, hematopoietic progenitors recruited to tumors to support their growth, [8, 9] correlate with primary prostate cancer progression. [10] While, these markers show promise in murine models, on cancer cell lines, and in primary tumor staining, they were not examined in the circulation of patients or actually linked to tumor presence. Further, the use of these markers in a diagnostic or prognostic capacity remains untested. To be effective for diagnosis, possible markers must be released into the circulation by the tumor at levels measurable above background. [11]

The recommended screening test for prostate cancer: prostate specific antigen (PSA) is mired in controversy surrounding its benefits and its ability to guide appropriate treatment options for patients with newly-diagnosed, localized prostate cancer. Partly, this is due to the favorable natural history of low-grade prostate cancer, but also to diagnostic imperfections in PSA and prostate biopsies. Current techniques are incapable of accurately predicting which patients will suffer future recurrence, regardless of their diagnosed pathologic stage and therapy. Recently, a PSA-based screening study demonstrated a 20% reduction of prostate cancer-specific death. [12] However, this required screening 1410 men and treating 48 additional cases to prevent one death. [12] These findings suggested high rates of over-diagnosis and over-treatment of clinically insignificant prostate cancer and stressed the need to develop new markers to effectively identify men at risk of dying from prostate cancer.

In this prospective study, we investigated circulating CD117^+^, CD133^+^, CXCR4^+^, and CD34^+^ cell levels in patients undergoing surgical treatment for localized prostate cancer. We designed an exploratory study to compare the levels of circulating cells of tumor origin in the same patient prior to and after tumor removal with additional follow up for recurrence. We found that only CD117 was a potential diagnostic marker for prostate cancer and that its expression levels were associated with EpCAM expression, PSA values, and future recurrence. Using a xenograft model, we demonstrated that CD117 expression enhances tumor growth and angiogenesis. Thus, CD117 may play a role in tumor development and its expression on circulating cells may function as a marker for residual or recurrent disease after primary therapy.

## RESULTS

### CD117^+^ circulating cells decrease after tumor removal

**Figure 1:**
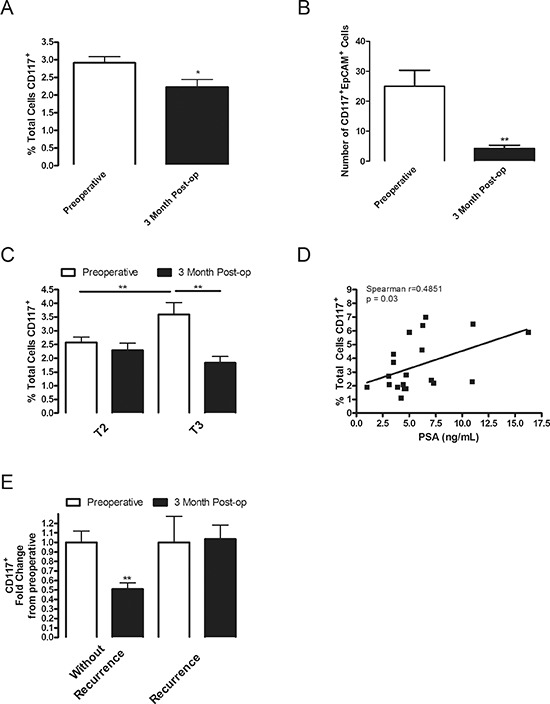
CD117 expression decreases after tumor removal, is dual-positive for EpCAM, increases in high-grade patients and remains elevated in patients with biochemical recurrence **(A)** Circulating lymphocytes were isolated from the whole blood of patients undergoing radical prostatectomy preoperatively (white columns; *n* = 115) and 3 months (black columns; *n* = 61) post-operatively (post-op) and stained for CD117 expression. Percentage of stained cells represented as mean ± SEM. **(B)** Circulating lymphocytes were isolated from patients undergoing radical prostatectomy preoperatively and dual stained with EpCAM and CD117. Numbers of positively dual stained cells represented as mean ± SEM. **(C)** Percentage of CD117 stained cells for patients under 60 years of age separated into T2 and T3 stages. **(D)** Percentage of CD117^+^ stained cells in T3 patients under 60 years of age were plotted in relation to their reported PSA value with a linear regression line shown (*n* = 19). **(E)** Compliant T3-staged patients were separated into groups without recurrence (*n* = 12) and those with a biochemical recurrence defined as a postoperative PSA above 0.02 ng/mL (*n* = 8). * represents *p* < 0.05 and ** represents *p* < 0.01 by one-way ANOVA.

Several cell surface markers, including CD133, CD117, CD34 and CXCR4 were proposed to reflect the presence and/or severity of tumors. To establish whether these markers are predictive for human cancers, numbers of CD133^+^, CD117^+^, CD34^+^, or CXCR4^+^ circulating cells were assessed in prostate cancer patients before and after prostatectomy. A total of 115 patients were recruited in the primary cohort and blood samples collected preoperatively (*n* = 115) and 1–3 months (*n* = 79) postoperatively (patient recruitment details are described in Materials and Methods). The clinical and demographic characteristics of the primary cohort are described in Supplementary Table 1. Of note, both CD117^+^ and CD133^+^ staining increased in primary cancerous cores relative to begin prostate tissue in 11 randomly selected patients (Supplemental Fig. 1), confirming previous data. [3, 5, 13] Frequencies of circulating CD117^+^ cells were significantly lower in postoperative relative to preoperative samples (Fig. 1A). However, percentages of circulating CD133^+^, CD34^+^, or CXCR4^+^ cells remained near preoperative levels (Supplemental Fig. 2). Thus the data suggest CD117^+^ cells represent a potential circulating diagnostic marker identifying prostate cancer.

### CD117^+^ circulating cells express the prostate tumor marker, EpCAM

Prostate tumor-derived cells express the surface marker EpCAM. [14, 15] In a secondary cohort of 16 patients the expression of EpCAM and CD117 was examined to establish whether CD117^+^ cells were also EpCAM^+^. The secondary cohort patient demographic and clinical parameters (Supplementary Table 1) and circulating marker levels were similar to the primary cohort. Approximately 20% of circulating cells in preoperative patients were CD117^+^EpCAM^+^ (Fig. 1B) suggesting a subset of tumor-derived circulating CD117^+^ cells. Interestingly this is approximately the same percentage of cells that are lost after tumor removal. As anticipated the numbers of CD117^+^EpCAM^+^ cells declined after prostatectomy (Fig. 1B).

### Increased CD117^+^ circulating cells are associated with higher PSA and tumor severity

In younger patients, circulating PSA levels have a predictive value, albeit controversial, of tumor progression. [16] To further determine the diagnostic utility of CD117, the frequencies of CD117^+^ circulating cells in patients under 60 years of age were compared to PSA levels and clinical risk stage. The primary cohort was separated into two groups: T2, low-risk (preoperative: *n* = 39; 1–3 m: *n* = 29) and T3, high-risk (preoperative: *n* = 19; 1–3 m: *n* = 12). The frequencies of CD117^+^ circulating cells were higher in preoperative T3- (3.6 ± 0.4%) compared with T2-stage patients (2.6 ± 0.2%) (Fig. 1C), supporting CD117 as a predictive marker of tumor progression in younger patients. Following prostatectomy, a significant decline in the percentage of CD117^+^ circulating cells was only noted in T3-stage patients (1–3 m: 1.8 ± 0.2%) (Fig. 1C). Notably, similar frequencies of CD133^+^ circulating cells were detected between preoperative and postoperative T2- and T3-stage patients (Supplemental Fig. 3A). Moreover, there was a positive correlation between PSA levels and percentages of CD117^+^ cells in T3-stage patients (Spearman *r* = 0.4851, *p* = 0.03; Fig. 1D), while CD133^+^ cells negatively correlated with PSA values (Spearman *r* = −0.3382, *p* = 0.04; Supplemental Fig. 3B).

### CD117^+^circulating cells remain elevated in patients with biochemical recurrence

During our study, eight compliant primary cohort patients (Supplementary Table 1) that were originally diagnosed with T3-stage tumors experienced biochemical recurrence subsequent to prostatectomy. To establish whether biochemical recurrence is associated with elevated CD117 expression, frequencies of CD117^+^ circulating cells in T3-stage patients with and without biochemical recurrence were compared. Interestingly, in contrast to “cured” patients, percentages of CD117^+^ cells did not decline in patients with biochemical recurrence (Fig. 1E). Notably, frequencies of CD133^+^ circulating cells were unchanged postoperatively, regardless of group (Supplemental Fig. 3C). These data indicate that postoperative circulating CD117^+^ cells may predict recurrence in high-risk patients.

### CD117 ligand stem cell factor (SCF) correlates with tumor severity

**Figure 2:**
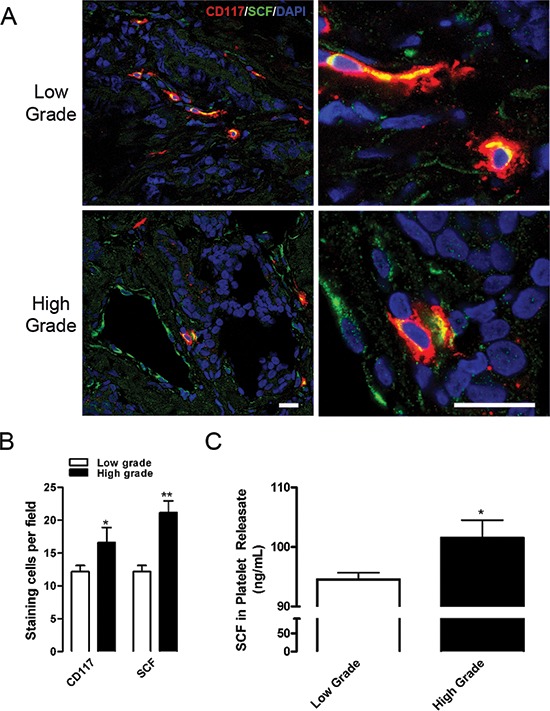
CD117 and SCF are expressed at higher levels in high-grade tumors **(A)** Representative prostate cancer sections from six low-grade (top panels) and six high-grade (bottom panels) tumors were stained for CD117 (red), stem cell factor (SCF, green), and nuclei (DAPI, blue). Scale bar = 20 μm. **(B)** Numbers of CD117 and SCF stained cells represented as mean number per field ± SEM (*n* = 6). **(C)** Platelet releasates were isolated from low-grade (white columns) and high-grade patients (black columns) and assayed for SCF concentration represented as mean ± SEM (*n* = 4–6). * represents *p* < 0.05 and ** represents *p* < 0.01 by Student's *t* test vs. low-grade.

CD117 binding to SCF results in cell survival, proliferation, and differentiation. [3, 4, 13, 17, 18] To determine whether CD117 expression in primary tumors was associated with the levels of its ligand stem cell factor (SCF), we stained sections from high and low-grade patients for CD117 and SCF (Fig. 2A). CD117 and SCF staining increased 1.4- and 1.7-fold, respectively, in high-grade patients compared with low-grade (Fig. 2B).

Previously, we demonstrated that tumor-derived proteins sequestered within platelets control tumor progression and pre-metastatic signaling. [8, 19, 20] To determine whether circulating SCF could be a stimulus for CD117^+^ migration, we isolated platelet releasates from patients in the primary cohort with low-grade and high-grade tumors and measured SCF concentrations by ELISA. The concentration of platelet SCF was significantly higher in high-grade patients compared with low-grade (Fig. 2C). Thus, circulating SCF may be acting as a potential stimulant promoting survival and proliferation of CD117^+^ cells entering the circulation.

### CD117 positive cells generate larger tumors with increased vascularization and proliferation

**Figure 3:**
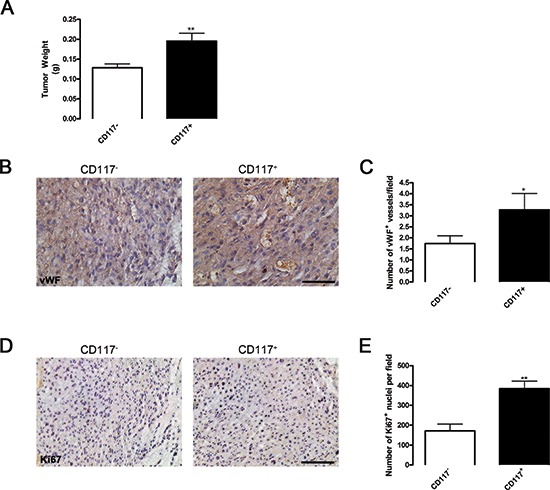
CD117 positive cancer cells generate larger tumors due to increased blood vessel formation **(A)** Human prostate cancer cells (LNCaP-C4–2) were sorted into CD117^−^ (white columns) and CD117^+^ (black columns) cell populations, mixed with matrigel, and implanted in SCID mice. After 30 days, tumor size was represented as mean weight ± SEM (*n* = 11–16). **(B)** Representative images of tumors sectioned and stained for mature vessels using von Willebrand factor (vWF). Scale bar = 50 μm. **(C)** The number of vWF^+^ vessels were represented as mean number per field ± SEM (*n* = 11–19). **(D)** Representative images of tumors sectioned and stained for cell proliferation using Ki67. Scale bar = 100 μm. **(E)** The number of Ki67^+^ cells were represented as mean number per field ± SEM (*n* = 5–11). *represents *p* < 0.05 and **represents *p* < 0.01 by Student's *t* test vs. CD117^−^.

To determine whether CD117 was involved in tumor progression, we analyzed several human prostate cancer cell lines for CD117 expression. CD117 levels were the highest on LNCaP-C4–2 cells (Supplemental Fig. 4A) and these cells were also weakly EpCAM positive (Supplemental Fig. 4B). LNCaP-C4–2 cells were sorted into CD117^+^ and CD117^−^ populations and implanted in immunocompromised mice. Tumors derived from the CD117^+^ population were 1.5-fold larger than CD117^−^ tumors (Fig. 3A). Tumors from the two populations were then stained for von Willebrand factor (vWF) to assess tumor vascularization (Fig. 3B) and Ki67 to measure cell proliferation (Fig. 3D). Quantification of vWF stained vessels demonstrated 1.9-fold more vessels in CD117^+^ tumors compared with CD117^−^ (Fig. 3C). In addition, quantification of Ki67^+^ nuclei showed that 2.2-fold more cells in CD117^+^ tumors were proliferating compared with CD117^−^ (Fig. 3E). These data demonstrate that CD117^+^ human prostate cancer cells display increased tumor growth and angiogenesis.

## DISCUSSION

In this study, we assessed the levels of prominent prostate cancer markers: CD117, CD133, CXCR4, and CD34, on circulating cells from prostate cancer patients before and after radical prostatectomy. Only CD117 expression increases in high-risk patients, while the other markers remained unchanged. Moreover, only CD117^+^ cell levels decrease substantially after prostatectomy, which, together with EpCAM positivity, confirm their tumor origin. Interestingly, the percentage of CD117^+^ cells that were EpCAM^+^ is exactly the same as the percentage of total CD117^+^ cells lost upon tumor removal, indicating that it is likely these cells of tumor origin that are absent in postoperative patients. In patients with biochemical recurrence, however, CD117 levels do not change after prostatectomy. CD117 and its ligand, SCF, are involved in tumor progression, since CD117^+^ cells form larger, more aggressive tumors in animal models. Our data suggest that CD117 on tumor cells is an important player in tumor dissemination and the CD117^+^ cell levels in the circulation may be predictive of locally advanced prostate cancers and biochemical recurrence.

We demonstrate that CD117^+^ cell levels directly correlated with PSA levels in younger patients. We also found that in patients with PSA values above 4 ng/mL CD117 expression was increased (data not shown). As PSA values above 4 ng/mL are used as a biopsy indicator; [21] circulating CD117 expression could be an additional minimally invasive test with PSA to identify patients likely to have locally advanced prostate cancer. While circulating CD117^+^ cell presence correlated directly with PSA levels, there was no direct correlation with any other demographic or clinical characteristics in any manner prognostic for prostate cancer. This lack of additional correlation does not diminish the value of these markers alone or in combination with currently described factors in predicting risk. In fact, other studies demonstrated increased survival in prostate cancer patients with CD117^−^ biopsies compared to patients displaying CD117 expression. [3] Further studies are required to determine the clinical utility of circulating CD117^+^ cell levels in prostate cancer diagnosis and staging.

Our xenograft studies demonstrate that CD117^+^ tumor cells form larger and more vascularized tumors compared to CD117^−^ cells. This suggests that CD117 is not only a marker of tumor progression and metastasis, but an important driver of these processes. Our study shows that CD117 staining was significantly elevated in prostate tumors from high-grade patients, compared with low-grade, corresponding with previous reports demonstrating higher staining in patient bone metastases compared with the primary tumor. [13] We demonstrate that both CD117 and SCF are elevated in high-grade tumors and often colocalize suggesting the presence of autocrine activation loop. Importantly, in patients with high-grade cancer, SCF was elevated in platelets which selectively uptake and may selectively release tumor-derived proteins. [19, 22] The activation of CD117 by SCF results in cell survival, proliferation, and differentiation. [23] SCF in the circulation or bone marrow [24] may serve as a chemotactic factor [23] stimulating the mobilization of CD117^+^ cells into the circulation. Further, exogenous SCF treatment promotes expression of CD117 by prostate cancer cells, in addition to the stem cell factors Oct3/4 and Nanog, [17] indicating that a stem cell like phenotype may be induced by CD117 activation by SCF.

Why certain cancer patients experience relapse after treatment for clinically localized disease is unknown. During the last decade, the concept of “cancer stem cells” (CSC) has emerged as one possible explanation for the initiation, progression, and relapse of tumors. [25] These CSCs constitute a small fraction of tumor cells and they possess unlimited self-renewal properties along with an ability to differentiate and induce phenotypic copy of the original tumor. [25, 26] Essentially, without CSCs, the tumor tissue would eventually degenerate. Interestingly, these cells may remain dormant for years and are resistant to cytotoxic therapies. [26] Furthermore, if prostate cancer metastasis is driven by this small population of CSCs, it might explain the failure to detect metastases by PSA screening and the failure to develop therapies that eradicate the initially “localized cancer.” In addition, CSCs may remain during the passaging of cancer cells lines. [27] Since CD117^+^ cells were only a subpopulation of the LNCaP-C4–2 cell line, they may represent a CSC which is maintained during passaging and retains the ability to initiate and promote tumorigenesis. Further, CD117^+^ osteosarcomas formed tissues upon implantation, while CD117^−^ cells lacked this ability. In addition, these tumor-initiating cells were highly invasive and resistant to common chemotherapeutic agents. [28] CD117^+^ tumors were more invasive, and when CD117 was activated, tumors initiated oncogenic signaling and gene transcription program resulting in further invasiveness. [6] In addition, using a xenograft model, we show that CD117 expression results in increased tumor growth due to enhanced angiogenesis and tumor proliferation. Further studies are required to characterize the CD117^+^ cells circulating in prostate cancer patient blood to define whether these cells represent CSCs, [25] CTCs, or another progenitor type supporting tumor growth and metastasis.

While only CD117 was a prostate cancer marker in our study, this does not mean that the other markers (CD133, CXCR4 and CD34) were not altered by tumor presence. Since CD133, CXCR4, and CD34 are found on bone marrow and hematopoietic progenitors, their changes could be masked by the high numbers already in the circulation, especially at early time points after surgery. Further, there may be utility in looking for dual expression of CD133 or CXCR4 with CD117 on circulating cells as co-expression of these markers are pro-tumorigenic in murine models. [4, 29] In summary, we demonstrate that circulating CD117^+^ cells are increased with cancer severity as assessed by stage and grade and may be a marker for locally advanced tumors. The ability to distinguish which patients are likely to progress to advanced disease or experience recurrence will prevent unnecessary surgeries and other interventions resulting in improved treatment outcomes. [12] Our data demonstrate that analysis of CD117^+^ cells in the circulation could be used in the diagnosis of prostate cancer, in developing prognosis, or to determine treatment efficacy.

## MATERIALS AND METHODS

### Patients and blood samples

Institutional Review Board approval and signed informed consent was obtained prior to initiating prospective blood sample collection. An initial sample size of 96 patients was required to obtain a power of 90% (NCSS 2007) based on an independent pilot study of 27 patients, in which the potential biomarkers were initially identified (data not shown). A total of 115 consecutive patients who underwent radical prostatectomy at the Cleveland Clinic between September 2008 and May 2010 participated in the study. Blood samples from these patients were used for marker identification and recurrence analysis. A second cohort of 16 patients was recruited between November 2011 and March 2012 and these samples were used for secondary analysis of CD117^+^ cells including dual staining. All patients presented with clinically localized prostate cancer without prior radiation and no additional forms of cancer. Patients were given standard care preoperatively and postoperatively and followed for disease recurrence with serum PSA determinations (biochemical recurrence: PSA ≥ 0.03 ng/mL postoperatively) and clinical assessment every 3 months.

Whole blood (3–4 mL) was collected prior to radical prostatectomy (preoperative group), then postoperatively at approximately 1–2 weeks (1wk group) and 1–3 months (3 m group). The definition for low-risk disease in this study consisted of localized prostate cancer with a final pathology of Gleason score 6–7, clinical stage (TNM) T1 (a,b,c) or T2 (a,b), and PSA level ≤ 10 ng/mL; high-risk disease included a Gleason score 8–10, clinical stage T2c or T3 (a,b), and PSA level > 10 ng/mL. Clinical parameters are described in Supplemental Table 1.

### Immunohistological staining

Paraffin-embedded prostate specimens were step-sectioned at 3 mm intervals and mounted as half-sections or quarter-sections for microscopic review by a genitourinary pathologist. A total of 11 patients were randomly selected (7 low-grade and 4 high-grade) from the primary cohort for immunohistological staining with CD117 (1:500, Dako) or CD133 (1:100, Cell Signaling). Hematoxylin and eosin (H&E) staining was completed on serial sections. Images were taken on a Leica DM2500 microscope. Ratio of stained area to total area was calculated for 3 fields per patient using ImagePro Plus 5.0 (Media Cybernetics). Staining was completed blinded to the study endpoint.

### Lymphocyte isolation and marker analysis

Lymphocytes were isolated from whole blood collected by venipuncture in Na_2_EDTA (VWR) tubes by density gradient centrifugation using Ficoll-Paque PLUS (GE Healthcare) according to the manufacturer's protocol. Pelleted lymphocytes were resuspended in DMEM/F12. Isolated lymphocytes were initially blocked with human FcR blocking reagent (1:100; Pierce) and subsequently incubated with fluorescently conjugated antibodies (1:50): anti-CD133/prominin-1-APC (Miltenyi Biotec), anti-CD184/CXCR4-PE (R&D Systems), anti-CD34-FITC (BioLegend), anti-CD117/c-kit-APC (Miltenyi Biotec) or anti-EpCAM/CD326-FITC (Miltenyi Biotec). Prior to analyzing lymphocytes with the BD FACS Canto II and the FACS Diva software (BD Biosciences), samples were fixed in 1% formalin. Fluorescence values of stained lymphocytes were normalized to an unstained control sample and initial compensation was applied using corresponding fluorescently conjugated IgG control antibodies. All flow cytometry was performed on de-identified samples directly after blood collection by a person blinded to the clinical characteristics of the patients. Marker expression on prostate cancer cell lines was measured as described above.

### Immunofluorescent staining

Frozen prostate cancer sections were obtained and stained with rabbit anti-SCF (1:100; Abcam) and mouse anti-CD117/c-kit (1:400; Cell Signaling) antibodies after fixation in 4% paraformaldehyde. After incubation with primary antibodies, samples were washed and exposed goat anti-rabbit Alexa Fluor488 and anti-mouse Alexa Fluor568 (Invitrogen) secondary antibodies. The slides were mounted with medium containing DAPI (Dako) and images were taken by a TCS-SP (Leica) microscope. For quantification, the images were analyzed with ImagePro software (Media Cybernetics).

### SCF ELISA

An aliquot (2 mL) of the whole blood was collected by venipuncture in Na_2_EDTA tubes from patients prior to radical prostatectomy (as described above) was used for platelet isolation. Platelets were separated from the platelet-rich plasma of whole blood by gel filtration, as previously described (Kerr et al, 2010) and activated as previously described (Kerr et al, 2013; Kerr et al, 2010). Isolated platelet releasates from 3 low-grade and 2 high-grade patients were assayed using the R&D Systems Quantikine Human SCF ELISA according to the manufacturer's instructions. Two separate samples from each patient were analyzed and compared to a standard curve to obtain the concentration in pg/mL of each protein in the samples. The assay and analysis was performed blinded to the study.

### Xenograft tumor implantation and analysis

Eight week old male NOD.CB17-*Prkdc^scid^*/J (Jackson Laboratory) mice were injected subcutaneously with 1.8 × 10^5^ sorted LNCaP-C4–2 human prostate cancer cells in 200 μL matrigel per side (*n* = 7–10). LNCaP-C4–2 cells were provided by Dr. Lloyd A. Culp (Case Western University) and were sorted into CD117^−^ and CD117^+^ populations based on staining with anti-CD117-APC gated based on a mouse IgG-APC isotype control on a BD FACSAriaII cell sorter by the LRI Flow Cytometry Core. Tumors were permitted to grow for 30 days before mice were sacrificed and tumors were removed and weighed. Tumors were fixed in 10% formalin for 2 hours then dehydrated and embedded in paraffin. Paraffin sections were stained for vWF (1:200; Dako) or Ki67 (1:50; Dako) and staining was visualized using the Vectastain Elite ABC kit (Vector Laboratories) and SIGMAFAST 3,3′-diaminobenzidine tablets. Images were taken on a Leica DM2500 microscope. The number of vWF stained vessels were counted in 3 fields per tumor (*n* = 9) in a blinded fashion. The number of Ki67 positive nuclei per field were counted using ImagePro Plus 5.0 (*n* = 5–11).

### Statistical analysis

Values are represented as mean ± SEM. Two tailed Student's *t* test analysis or one-way ANOVA analysis with Newman-Keuls post-test determined statistical significance. Spearman correlations revealed relationships between marker expression and clinical parameters. All statistical analysis was completed using GraphPad Prism 5.0 software.

## SUPPLEMENTARY FIGURES AND TABLE


