# Supplementary material for: CD117+ cells in the circulation are predictive of advanced prostate cancer
Source: Oncotarget. 2014 Dec 17;6(3):1889–97. doi: 10.18632/oncotarget.2796 (PMC4359340; doi:10.18632/oncotarget.2796)
Supplement: Supplementary file 1 [file oncotarget-06-1889-s001.pdf]

SUPPLEMENTARY FIGURES AND TABLE

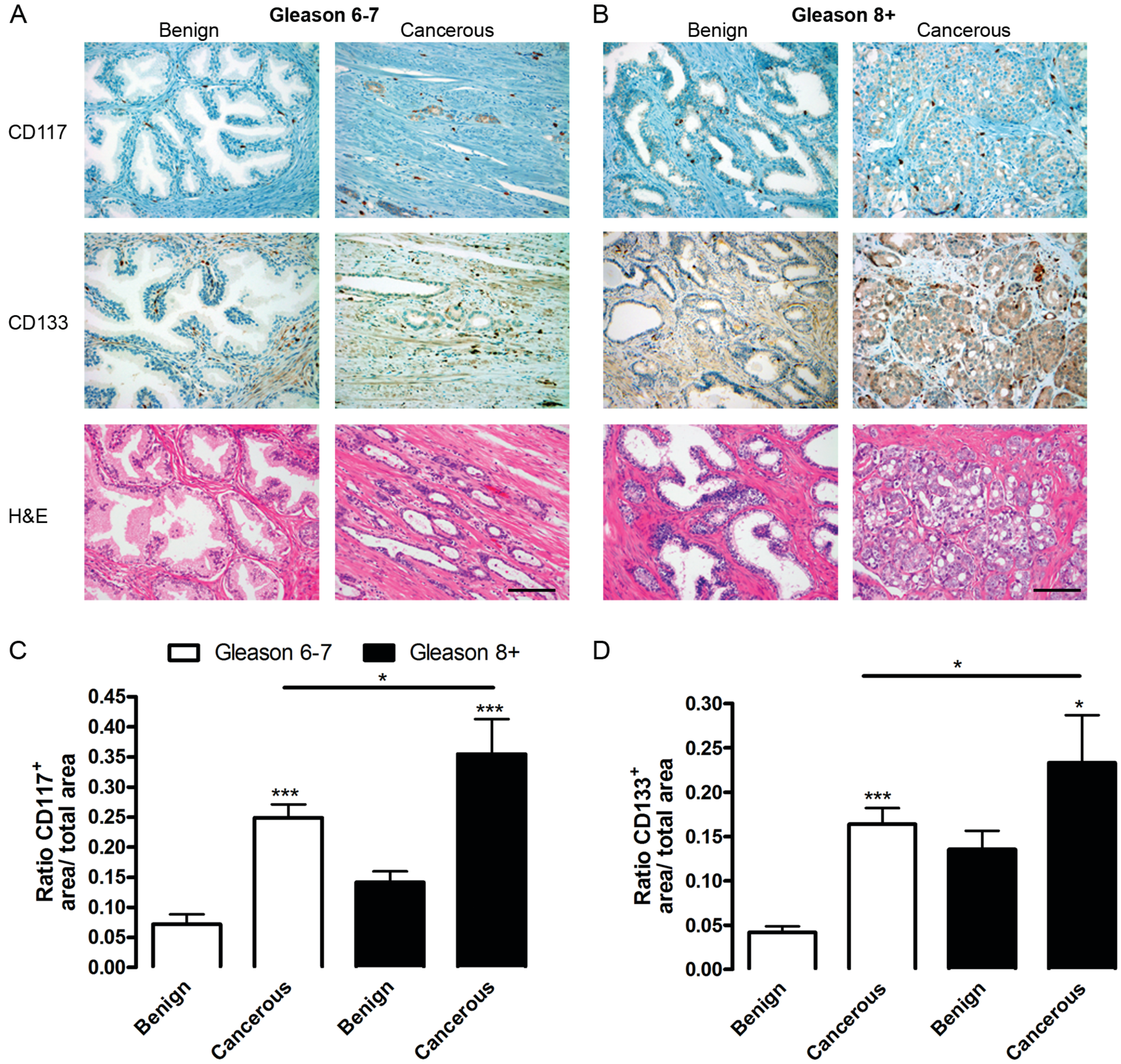

Supplementary Figure 1: CD117 and CD133 expressions are increased in high-grade primary prostate tumors. Sections of primary prostate cancerous and benign areas from the same patient were stained for CD117 (1:500, Dako, top), CD133 (1:100, Cell Signaling, middle), or H&E (bottom). Patients were grouped by Gleason score 6–7 (left panels, white columns) or over 8 (right panels, black columns). Images are representative of 7 low-grade **(A)** and 4 high-grade patients **(B)**. Scale bar represents 100 µm. Staining was quantified for CD117 **(C)** and CD133 **(D)** using Image Pro and represented as the ratio of stained area to total area for each field ± SEM. * represents $p < 0.05$ and *** represents $p < 0.005$ by one-way ANOVA.

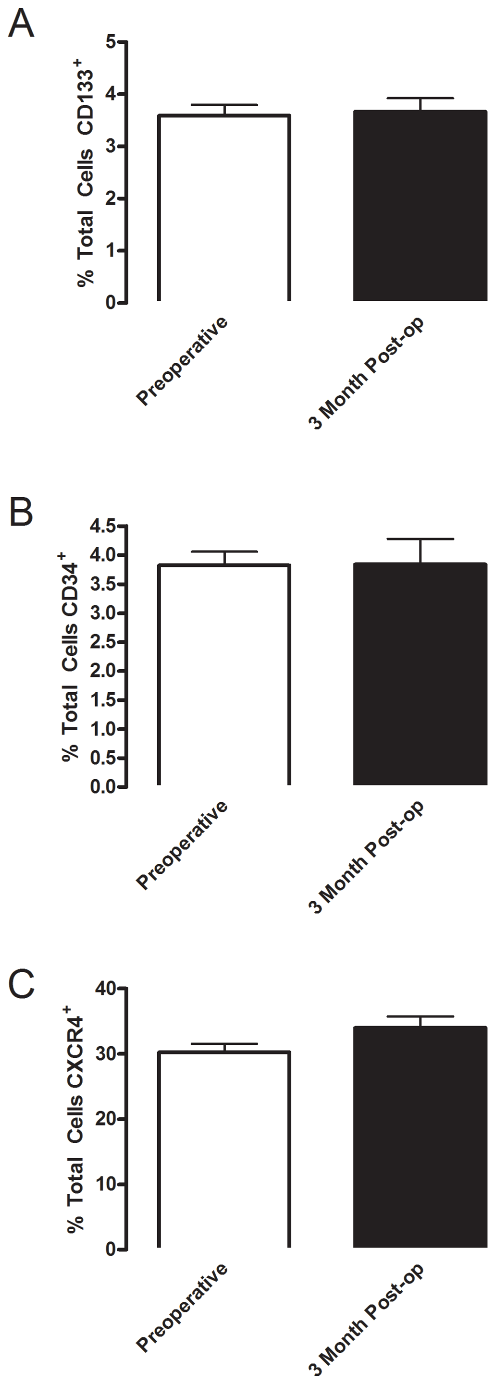

Supplementary Figure 2: Other marker expression does not change after tumor removal. **(A–C)** Circulating lymphocytes were isolated from the whole blood of patients undergoing radical prostatectomy preoperatively (white columns; $n = 115$) and 3 months (black columns; $n = 61$) post-operatively (post-op) and stained for CD133 (A), CD34 (B), and CXCR4 (C) expression. Percentage of stained cells represented as mean ± SEM. * represents $p < 0.05$ and ** represents $p < 0.01$ vs. preoperative by one-way ANOVA.

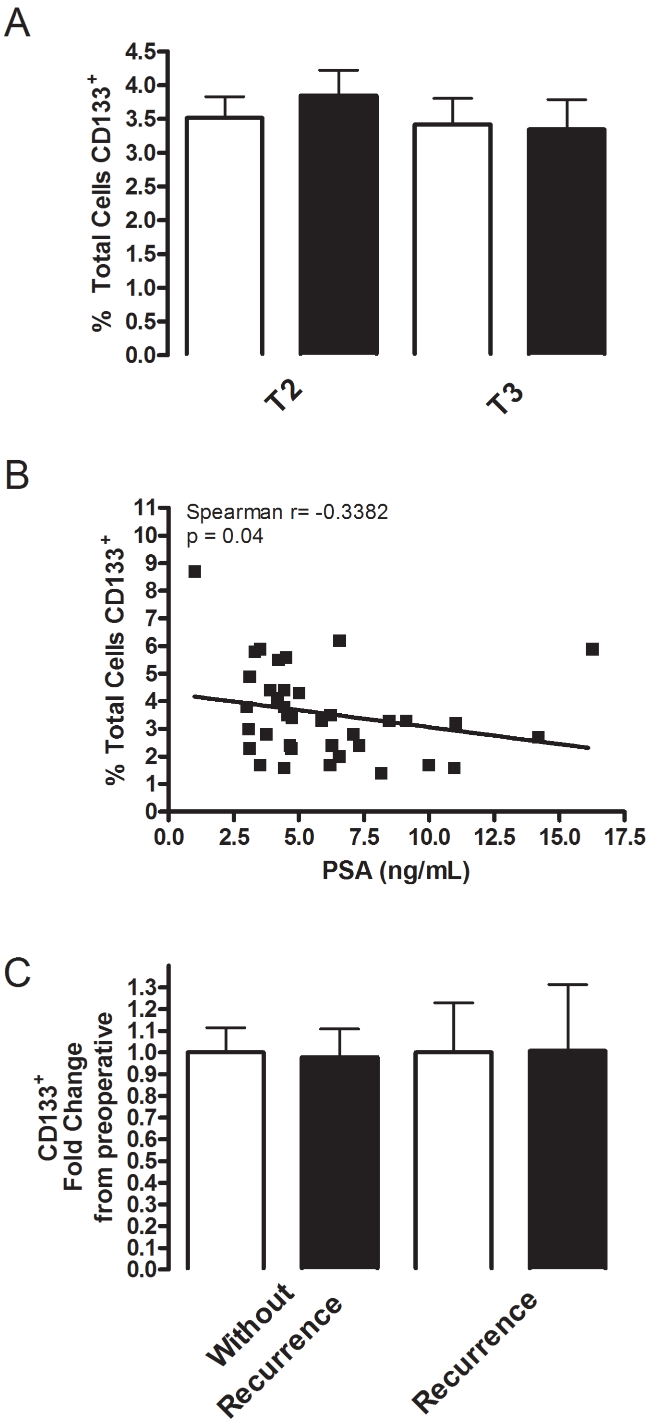

Supplementary Figure 3: CD133 expression is constant with increased stage and recurrence. Circulating lymphocytes were isolated from the whole blood of patients undergoing radical prostatectomy preoperatively (white columns) and 3 months (black columns) post-operatively (post-op) and stained for CD133 expression. Percentage of stained cells represented as mean ± SEM. **(A)** Percentages for patients under 60 years of age separated into T2 and T3 stages. **(B)** Percentage of CD133+ stained cells in T3 patients under 60 years of age were plotted in relation to their reported PSA value with a linear regression line shown (n = 19). **(C)** Compliant T3-staged patients were separated into groups without recurrence (n = 12) and those with a biochemical recurrence defined as a postoperative PSA above 0.02 ng/mL (n = 8). * represents p < 0.05 and ** represents p < 0.01 by one-way ANOVA.

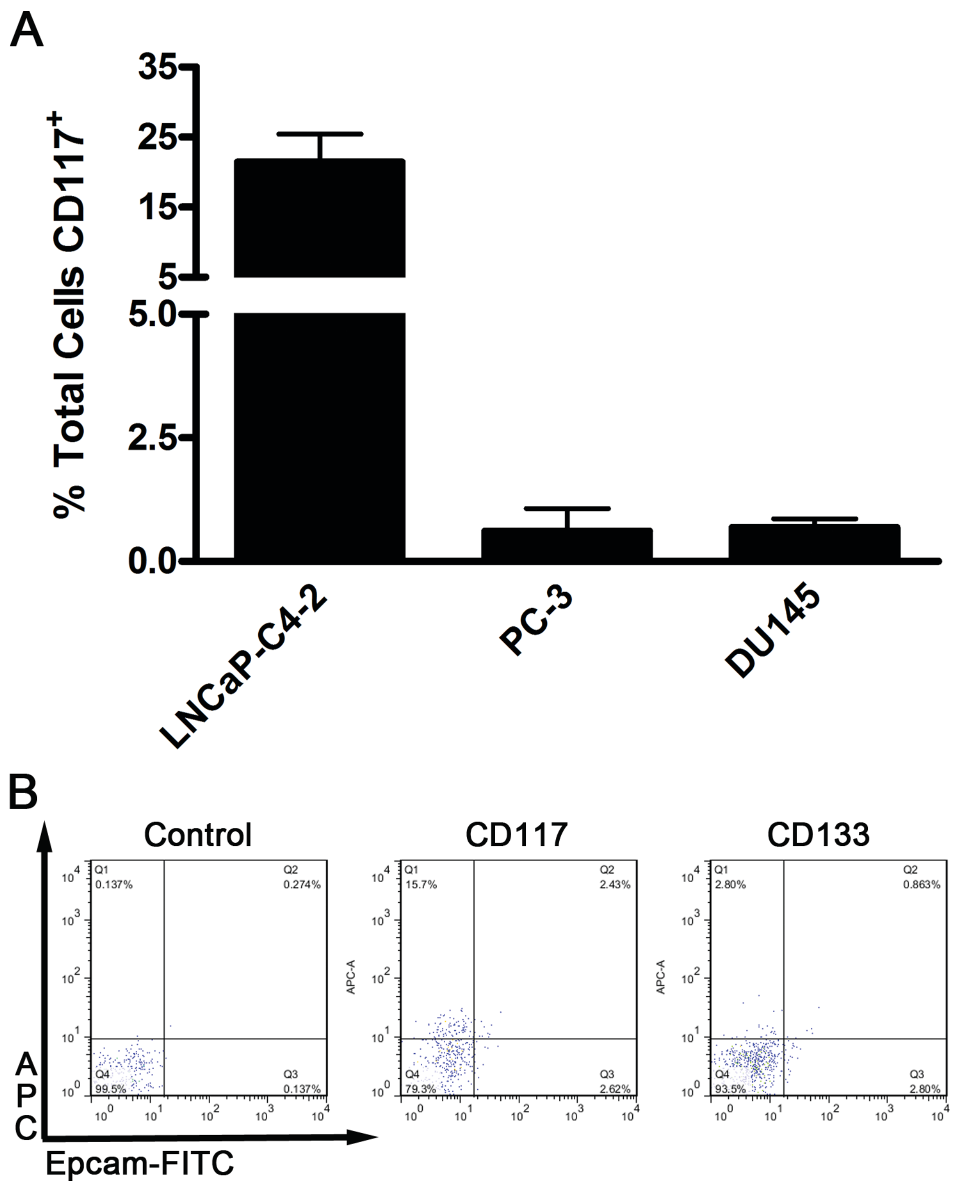

Supplementary Figure 4: CD117 is expressed in a variety of prostate cancer cell lines. **(A)** Prostate cancer cells lines were stained for CD117 expression represented as mean percentage of stained cells ± SEM (*n* = 2–5). **(B)** LNCaP-C4–2 cells were dual stained for EpCAM and CD117 or CD133. Isotype controls were used. Representative scatter plots of 3 experiments are shown.

Supplementary Table 1: Patient demographic and clinical parameters

	Primary Cohort (2008–2010)	Secondary Cohort (2011–2012)
Patients Recruited (n)	115	16
Age (yrs):		
Range	45 – 75	43 – 70
Mean ± SEM	58.41 ± 0.57	57 ± 1.77
Median	58	57
Hormone treatment		
Yes	3	0
No	102	16
NA	10	
PSA (ng/mL)		
Range	1.00 – 46.08	1.16 – 11.21
Mean ± SEM	6.13 ± 0.47	5.44 ± 0.67
Median	4.70	5.79
Tumor Volume (mm^3)		
Range	8 – 672	NA
Mean ± SEM	183.10 ± 13.71	NA
Median	150	NA
Pathological Gleason Grade		
3 + 3	28	0
3 + 4	58	14
4 + 3	15	2
4 + 4	5	0
4 + 5	7	0
Clinical Tumor Stage		
T2a	8	3
T2b	2	0
T2c	56	11
T3a	33	2
T3b	5	0
NA	12	0
Biochemical Recurrence		
Compliant Patients	8	NA
Total Patients	12	NA

NA represents data not available.
